# Substance Use and Cardiac Implantable Electronic Devices: A Single Institution Analysis of Re-Hospitalization, Follow-Up Rate, and Complications to Predict Patient Outcomes

**DOI:** 10.7759/cureus.99049

**Published:** 2025-12-12

**Authors:** Abby Stoecker, Paul Kang, Michael D White

**Affiliations:** 1 College of Medicine, Creighton University School of Medicine, Phoenix, USA; 2 School of Medicine, Creighton University School of Medicine, Phoenix, USA; 3 Cardiology, Creighton University School of Medicine, Omaha, USA

**Keywords:** cardiac implantable electronic device (cied), implantable cardioverter defibrillator, lead endocarditis, pacemaker, substance use

## Abstract

Introduction

Illicit drug use is a prevalent issue in Phoenix, Arizona, and is associated with various health concerns, including disrupting the conduction system of the heart. Cardiac implantable electronic devices (CIEDs), such as pacemakers and cardioverter-defibrillators, are common treatment options for conduction abnormalities. However, there is ongoing debate regarding the implantation of CIEDs in patients reporting active substance use, hypothesized to stem from provider concerns around lead endocarditis and poor follow-up adherence. Currently, information in the literature regarding outcomes of CIED patients with active substance use is lacking. This study aims to bridge this gap through evaluating differences in rehospitalization, follow-up, and CIED-related complications among patients with and without current substance use.

Methods

Patient records between January 2020 and December 2024 of those who received care at Valleywise Health in Phoenix, Arizona, were reviewed. All patients with complete records who had a CIED were included in the study. Covariates included patient demographics, substance use status, comorbidities, problems with CIED (infection, malfunction, adjustment/reprogramming), number of encounters, rehospitalization rates at 30 days, 90 days, and one year, and office follow-up rates. Continuous variables were compared between substance use statuses using the independent T-test or the Wilcoxon Rank Sum. Categorical variables were compared using the chi-squared analysis. Odds ratios (95% CI) were calculated using logistic regression to assess the association between substance use status, rehospitalization, and office follow-up while adjusting for potential confounding variables. Logistic regression modeling was repeated following a propensity score match to create comparable cohorts between patients with and without current substance use.

Results

A total of 2,522 patients were included in the study, with 90.4% reporting no current substance use and 9.64% reporting current substance use. There were no differences in 30-day, 90-day, and one-year rehospitalizations between patients with current substance use and patients with no substance use. There was also no difference in follow-up rates between these groups. In the unmatched analysis, Hispanic/Latino patients were less likely to have 30-day, 90-day, and one-year rehospitalizations compared to non-Hispanic/Latino patients. In the matched analysis, this was no longer significant. Additionally, in the unmatched analysis, patients reporting no substance use had more device malfunctions, adjustments, and infections compared to patients with current substance use. This difference was not seen following the propensity score match. Finally, patients who were unhoused were 23-fold (unmatched analysis) and 33-fold (matched analysis) more likely to have office follow-ups compared to housed patients.

Conclusion

This study demonstrated a lack of statistical difference in rehospitalizations and follow-ups between patients with CIEDs with and without substance use. This study also demonstrated that patients with CIEDs and current substance use were not at an increased risk for device infections or malfunctions compared to patients without current substance use. Further studies are necessary with larger populations to validate these conclusions.

## Introduction

The development of pacemakers and implantable cardioverter-defibrillators (ICDs) has expanded treatment options and quality of life for patients experiencing cardiac rhythm disturbances. Patients with the highest indications for pacemaker implantation include patients with AV nodal block and sick sinus syndrome, whereas ICDs are indicated for patients at risk for ventricular arrhythmias and sudden cardiac death, encompassing conditions such as ischemic/non-ischemic cardiomyopathy, long QT syndrome, and hypertrophic cardiomyopathy [1].

Additionally, illicit drug use can also increase the risk of developing cardiac arrhythmias [2]. Specifically, stimulants such as cocaine and methamphetamine can trigger tachyarrhythmias, with prolonged use resulting in myocyte cell death, thereby increasing the risk for future arrhythmias [2]. Given the established efficacy of cardiac implantable electronic devices (CIEDs), such as pacemakers and cardioverter-defibrillators, in managing rhythm abnormalities, it could be hypothesized that CIEDs would be similarly effective in patients with active substance use and coexisting rhythm disorders. However, there lack of consistent data regarding treatment patterns for patients with rhythm disorders and active substance use, as well as limited data around post-CIED outcomes in this population. For example, it has been reported that among patients presenting with stimulant-related cardiomyopathy, only 14% to 33% were treated with an ICD, with the majority receiving treatment geared towards drug rehabilitation/recovery [2]. However, it has also been reported that an additional percentage of patients may receive CIED therapy at follow-up after initial hospitalization [3].

The variability in CIED utilization in patients with active substance use has been hypothesized to reflect provider concern around follow-up adherence due to potential psychiatric illness, housing instability, or limited social support [2,4]. There is also reported concern that CIED patients with active substance use could be at an increased risk for developing lead endocarditis through intravenous drug use [2,4]. Although case reports have described instances of CIED lead endocarditis from intravenous drug use, observational studies analyzing outcomes such as rehospitalization, follow-up adherence, and device-related complications among CIED patients with active substance use remain limited [5,6].

An area that is disproportionately affected by substance use is Phoenix, Arizona, one of the top 10 cities in the United States with the highest rate of illicit drug use [7]. To gain more insight into the outcomes of CIED patients with active substance use, this study analyzed trends around follow-up rate, rehospitalization rate, and complications in CIED patients with and without current substance use at a Phoenix county hospital.

## Materials and methods

Data source

Data were collected from patient records between January 2020 and December 2024 at Valleywise Health Medical Center in Phoenix, Arizona. Valleywise Health is designated as a safety net hospital, with 14% of the patient population being uninsured, 15% living below the poverty level, and 54.9% identifying as Hispanic [8]. Between 2023 and 2025, 1,272 patients were diagnosed with a substance-related disorder (no tobacco), with alcohol and substance abuse being ranked as the first and third greatest community health conditions in 2019 and 2021, respectively [8].

Study population

All patients with a CIED and complete records who received care at Valleywise Health between January 2020 and December 2024 were included in the study. Patients with a CIED were identified using the ICD-10 codes Z95.810 (presence of automatic cardiac defibrillator with synchronous cardiac pacemaker, cardiac resynchronization therapy, or cardioverter defibrillator) and Z95.0 (presence of cardiac pacemaker). Patients reporting current substance use disorder (SUD) were identified with ICD-10 codes F11.10 (opioid abuse, uncomplicated), F14.10 (cocaine abuse, uncomplicated), F15.10 (other stimulant abuse, uncomplicated), F13.10 (sedative, hypnotic, or anxiolytic abuse, uncomplicated), and F19.10 (other psychoactive substance abuse, uncomplicated).

Demographics and covariates

Patient demographics such as age, biological sex (male, female, or unknown), ethnicity (non-Hispanic/Latino, Hispanic/Latino, or other), state of residence (Arizona or non-Arizona), and housing status (unhoused vs. housed) were collected. Covariates collected included "any heart disease" (ICD-10 codes 125.10, 125.110, 125.119, 125.84, 125.82), "any device malfunctions" (ICD-10 codes T82.118A, T82.897A), device adjustments (reprogramming)/management (ICD-10 codes Z45.02, T82.128A), and infection of the device (ICD-10 codes T82.7XXA). The percent tobacco risk from low, medium, and high was included as a patient demographics, as well as the mean number of encounters.

Outcomes

Patient outcomes were assessed by analyzing the percentage of 30-day, 90-day, and one-year rehospitalizations as well as the percentage of office follow-ups.

Statistical analysis

Patient demographic and baseline clinical characteristics between patients with and without current substance use were reported as means and standard deviations for normally distributed continuous variables following a histogram assessment. If a non-normal distribution was shown, medians and interquartile ranges were reported. The independent T-test compared normally distributed continuous variables between the groups, while the Wilcoxon Rank Sum test assessed non-normal continuous variables. Frequencies and percentages were reported for categorical variables, followed by chi-squared analysis or Fisher’s exact test to assess differences in categorical variables in relation to substance use status. Logistic regression calculated odds ratios and 95% confidence intervals to ascertain the association between substance use status and the odds of 30-day, 90-day, and one-year rehospitalization, respectively. As a secondary analysis, logistic regression assessed the association between substance use status and the likelihood of office follow-ups. All models were adjusted for age, sex, race, ethnicity, state of residence, unhoused status, primary diagnosis, tobacco risk, and the number of patient encounters. Logistic regression modeling was repeated following a propensity score match to create comparable cohorts between patients with and without current substance use. Propensity scores were estimated for substance use using age, sex, race, ethnicity, state of residence, housing status, primary diagnosis, tobacco risk, and the number of patient encounters. Finally, missing data were not considered random; thus, all analyses were conducted using the “Complete Case” approach to handling missing data. P-values were two-sided, and p < 0.05 was considered statistically significant. All data analysis was conducted using STATA version 18 (StataCorp: College Station, TX).

## Results

A total of 2,522 patients were included in the study, with 90.4% (n = 2,279) reporting no current substance use and 9.64% (n = 243) reporting current substance use (Table 1). Patients with substance use observed a lower mean age (57.4 + 12.5 vs. 68.1 + 15.1 years; p < 0.001) and a higher proportion of males (76.2% vs. 59.8%; p < 0.001) compared to patients without substance use. Furthermore, a lower percentage of Hispanic/Latino patients reported substance use (27.6% vs. 39.6%; p = 0.002). Overall, 98.1% of the patients resided in Arizona, and 39.1% reported being unhoused. While not statistically significant, a noticeable percent increase in Arizona residents was observed in patients with substance use (99.6% vs. 97.9%; p = 0.073), while a lower percentage of unhoused patients was reported within the same group (34.2% vs. 39.6%; p = 0.097). Among clinical covariates, a statistically significantly larger number of encounters was observed in patients with substance use, with a median (IQR) of 8 (3, 25) encounters compared to 6 (2, 21) encounters in patients without substance use (p = 0.001). Finally, patients without substance use reported a larger percentage of device malfunctions, device adjustments, and heart disease compared to patients with substance use (p < 0.001). Following the propensity score match, all demographic and clinical covariates were not statistically different between the two groups.

**Table 1:** Demographic and Clinical Characteristics Between Patients With Current Substance Use (SUD) vs. Patients Without Current Substance Use Continuous variables were compared using an independent T-test or Wilcoxon Rank Sum. Categorical variables were compared using chi-squared analysis.

		Unmatched			Matched		
Variables	Overall	No SUD	Yes SUD	p-value	No SUD	Yes SUD	p-value
Number of Patients	2,522	2,279 (90.4%)	243 (9.64%)		243 (50%)	243 (50%)	
Age, years (mean, SD)	67.1 (15.2)	68.1 (15.1)	57.4 (12.5)	< 0.001	57.9 (12.7)	57.4 (12.5)	0.64
Sex (n, %)							
Male	1,547 (61.3)	1,362 (59.8)	185 (76.1)	< 0.001	186 (76.5)	185 (76.1)	0.92
Female	974 (38.6)	916 (40.2)	58 (23.9)		57 (23.5)	58 (23.9)	
Unknown	1 (0.04)	1 (0.04)	0 (0.0)		0 (0.0)	0 (0.0)	
Ethnicity (n, %)							
Non-Hispanic/Latino	1,545 (61.3)	1,374 (60.3)	171 (70.4)	0.002	177 (72.8)	171 (70.4)	0.7
Hispanic/Latino	947 (37.6)	880 (38.6)	67 (27.6)		63 (25.9)	67 (27.6)	
Other	30 (1.19)	25 (1.10)	5 (2.06)		3 (1.23)	5 (2.06)	
State (n, %)							
Arizona	2,474 (98.1)	2,232 (97.9)	242 (99.6)	0.073	243 (100.0)	242 (99.6)	0.32
Non-Arizona	48 (1.90)	47 (2.06)	1 (0.41)		0 (0.0)	1 (0.41)	
Unhoused (yes, %)	986 (39.1)	903 (39.6)	83 (34.2)	0.097	82 (33.7)	83 (34.2)	0.92
Diagnosis (n, %)							
None	1,419 (56.3)	1,301 (57.1)	118 (48.6)	< 0.001	123 (50.6)	118 (48.6)	0.9
Any Heart Disease	913 (36.2)	821 (36.0)	92 (37.9)		90 (37.0)	92 (37.9)	
Any Device Malfunction	34 (1.35)	25 (1.10)	9 (3.70)		6 (2.47)	9 (3.70)	
Device Adjustments/Management	138 (5.47)	116 (5.09)	22 (9.05)		23 (9.47)	22 (9.05)	
Infection	18 (0.71)	16 (0.70)	2 (0.82)		1 (0.41)	2 (0.82)	
Tobacco Risk (n, %)							
Low	476 (18.9)	448 (19.7)	28 (11.5)	< 0.001	30 (12.4)	28 (11.5)	0.58
Medium	249 (9.87)	219 (9.61)	30 (12.4)		26 (10.7)	30 (12.4)	
High	163 (6.46)	125 (5.48)	38 (15.6)		29 (11.9)	38 (15.6)	
Missing	1,634 (64.8)	1,487 (65.3)	147 (60.5)		158 (65.0)	147 (60.5)	
Number of Encounters (median, IQR)	6 (2, 22)	6 (2, 21)	8 (3, 25)	0.001	8 (2, 22)	8 (3, 25)	0.51

**Figure 1:**
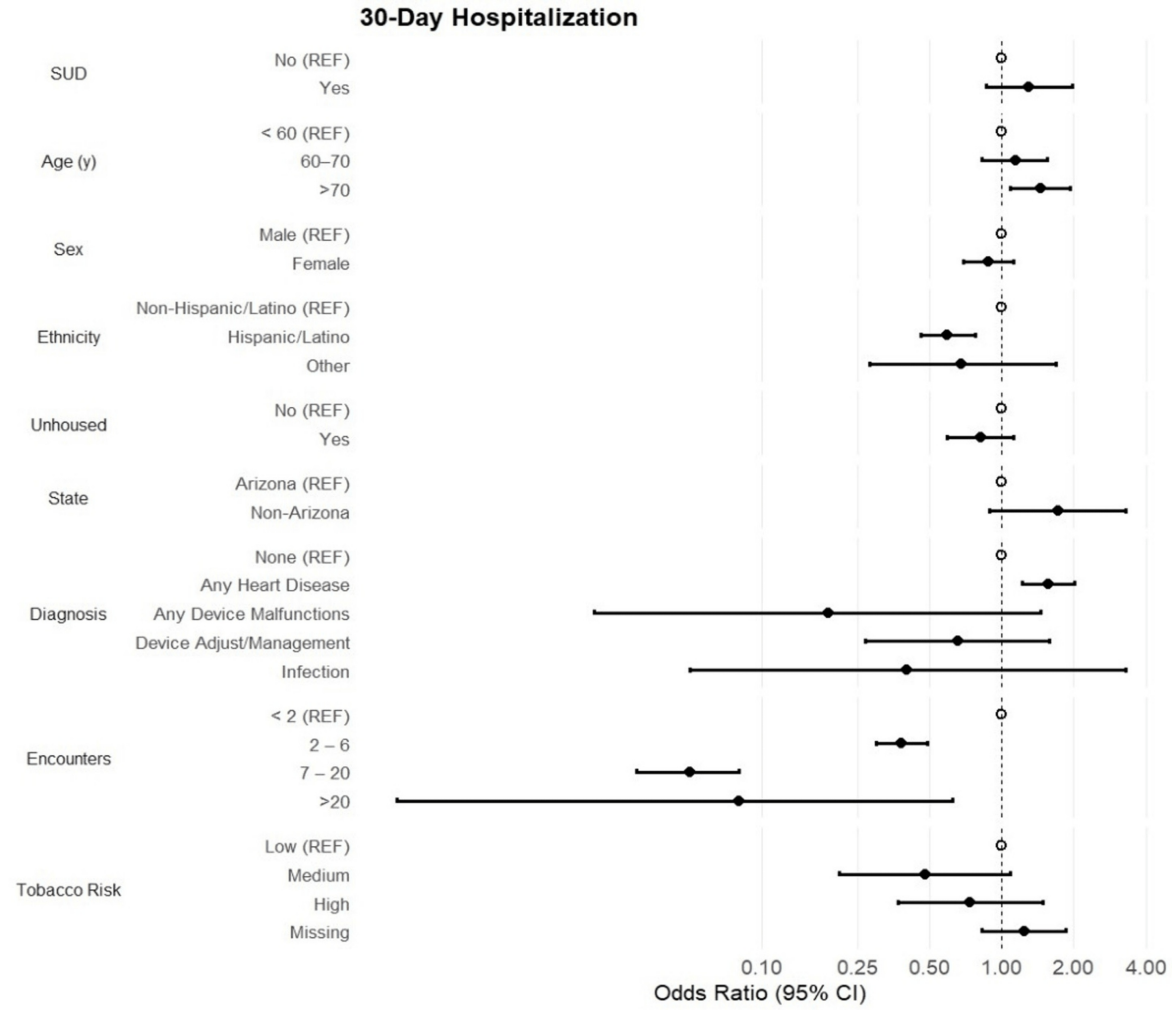
30-Day Hospitalization Forest plot of odds ratios and 95% CI assessing the association between substance use (SUD) and demographic and clinical characteristics with the odds of 30-day rehospitalization.

**Figure 2:**
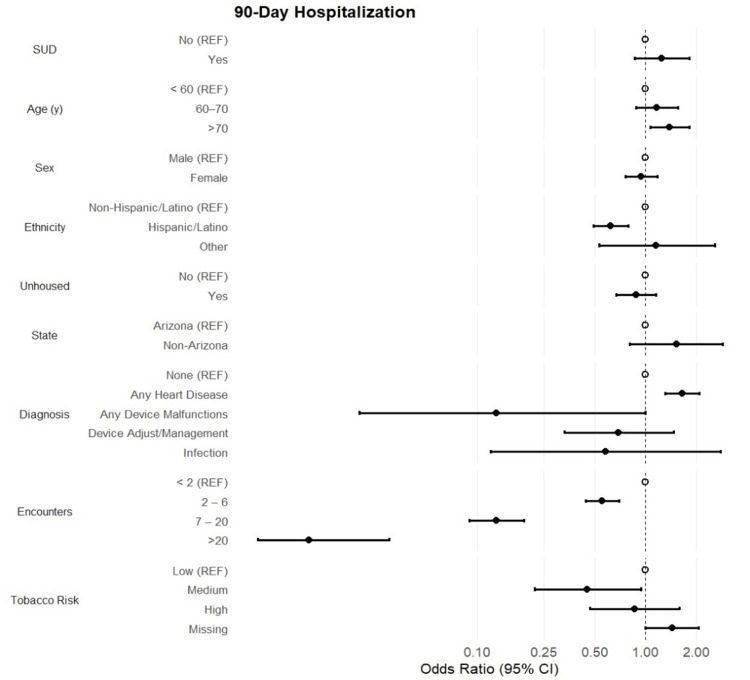
90-Day Hospitalization Forest plot of odds ratios and 95% CI assessing the association between substance use (SUD) and demographic and clinical characteristics with the odds of 90-day rehospitalization.

The overall percentage of 30-day and 90-day hospitalizations was 20.9% and 25.4%, respectively. No statistical associations between substance use status and 30-day (OR [95% CI] = 1.30 [0.86, 1.97]; p = 0.22) or 90-day hospitalizations (OR [95% CI] = 1.25 [0.86, 1.83]; p = 0.25) were reported (Figures 1, 2). Patients aged >70 years showed a 45% and 39% increase in the odds of 30-day and 90-day hospitalizations, respectively (p = 0.012). However, Hispanic/Latino patients reported 41% decreased odds of 30-day hospitalizations, while the odds of 90-day hospitalizations decreased by 38% among the same group compared to non-Hispanic/Latino patients (OR [95% CI] = 0.62 [0.49, 0.79]; p < 0.001). Patient sex and state of residence were not associated with rehospitalizations. Among primary diagnoses, patients with heart disease had 56% increased odds of 30-day hospitalization, with a 65% increased likelihood of 90-day hospitalizations (OR [95% CI] = 1.65 [1.31, 2.08]; p < 0.001). The frequencies of hospitalizations were rare among patients with device malfunctions or device adjustments; thus, no statistically significant associations were reported among these diagnoses (p > 0.05). Finally, the number of encounters was highly protective against 30- and 90-day hospitalizations. Among 30-day hospitalizations, odds decreased steadily as the categories of encounters increased, ranging from a 62% decrease in odds within the two-to-six encounters category (p < 0.001) to a 99% decrease in odds within the >20 encounters category compared to patients with fewer than two encounters. Similar trends are observed among 90-day hospitalizations. 

**Figure 3:**
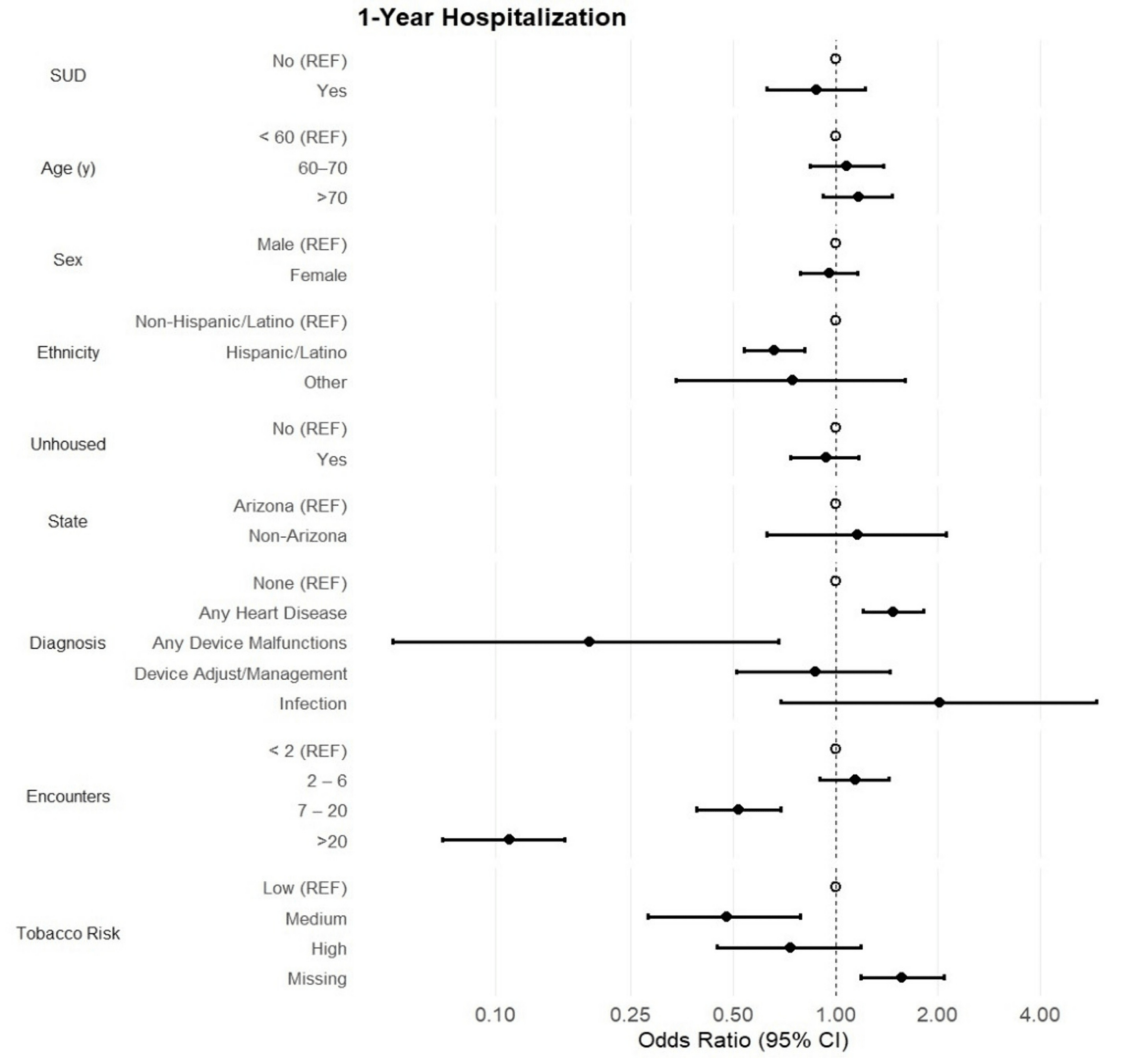
1-Year Hospitalization Forest plot of odds ratios and 95% CI assessing the association between substance use (SUD) and demographic and clinical characteristics with the odds of one-year hospitalization.

**Figure 4:**
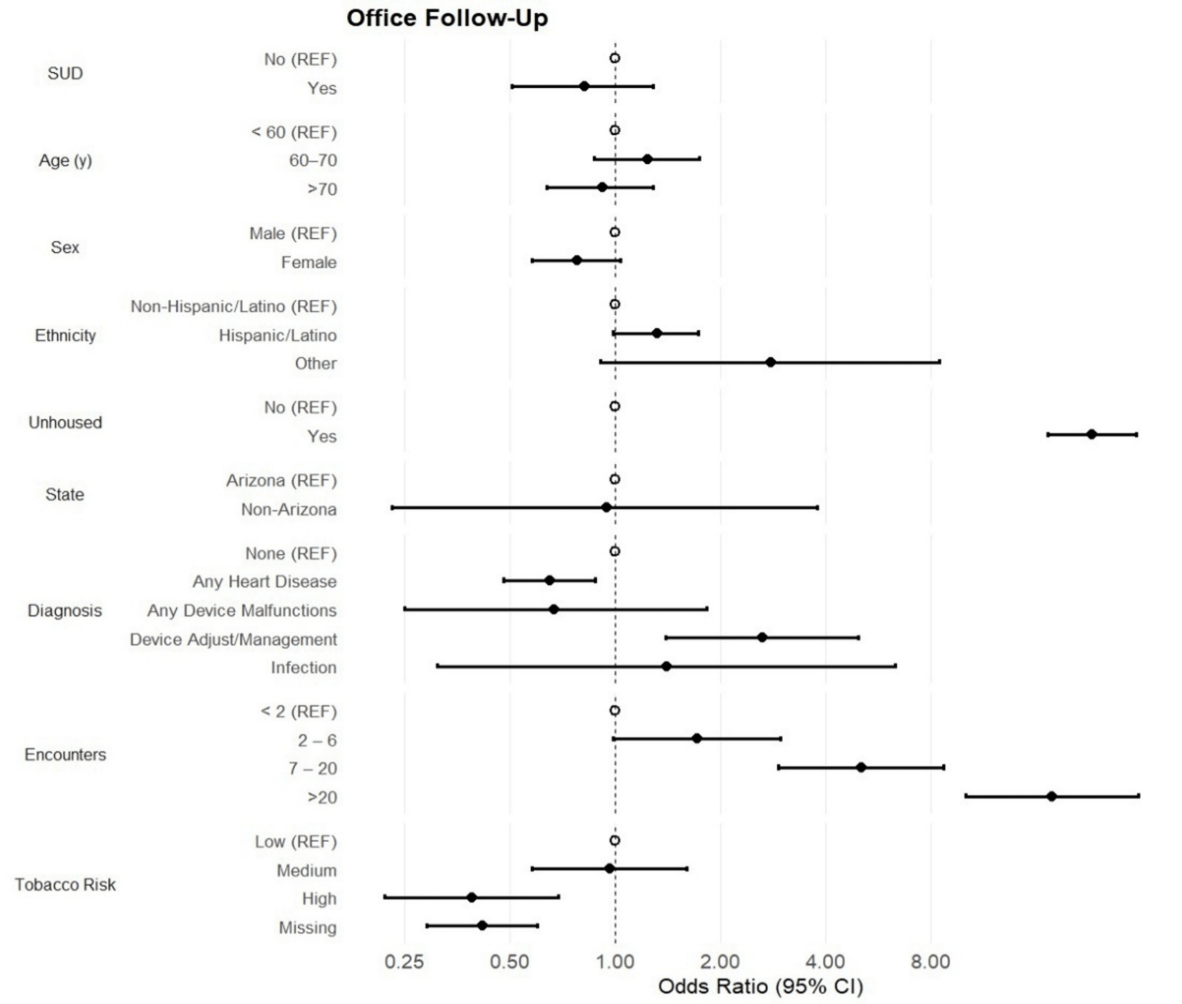
Office Follow-Up Forest plot of odds ratios and 95% CI assessing the association between substance use (SUD) and demographic and clinical characteristics with the odds of office follow-up.

Patients hospitalized within one year of their initial hospitalization accounted for 36.9% of the overall patient population. There was no statistically significant association between substance use status and one-year hospitalization (OR [95% CI]: 0.88 [0.63, 1.22]; p = 0.45) (Figure 3). Furthermore, patient age and sex were not found to be associated with the outcome. However, Hispanic/Latino status continued to show decreased odds of one-year hospitalization (OR [95% CI]: 0.66 [0.54, 0.81]; p < 0.001) compared to non-Hispanic/Latino patients. Patient diagnoses showed 48% increased odds of one-year hospitalizations among patients with any heart disease. Conversely, patients with any device malfunctions showed an 81% decreased likelihood of one-year hospitalization (OR [95% CI]: 0.19 [0.05, 0.68]; p = 0.011). The odds of one-year hospitalization decreased steadily as the categories of encounters increased. However, the negative association was not observed until patients had 7-20 encounters (OR [95% CI]: 0.52 [0.39, 0.69]; p < 0.001). With regard to office follow-up visits, no statistically significant association between substance use status and office visits was observed (OR [95% CI]: 0.22 [0.51, 1.29]; p = 0.39) (Figure 4). However, patients who reported being unhoused were 23-fold more likely to be seen for an office follow-up visit (OR [95% CI]: 23.1 [17.3, 30.9]; p < 0.001). Patients with heart disease were less likely to attend a follow-up visit, but those who required device adjustments were approximately three times more likely to attend a follow-up office visit (OR [95% CI]: 2.64 [1.40, 4.95]; p = 0.003). Finally, the odds of an office visit steadily increase as the number of encounters increases, ranging from 72% increased odds among patients with 2.6 encounters to a 17.8-fold increase among patients with >20 encounters (p < 0.001).

Within the matched cohort, substance use remained a statistically insignificant predictor with the odds of all outcomes (Table 2). However, the number of encounters was observed to have a negative association with hospitalization. Patients with greater than six encounters experienced a greater than 90% decreased odds of 30-day (OR [95% CI]: 0.02 [0.005, 0.006]; p < 0.001) and 90-day hospitalization (OR [95% CI]: 0.04 [0.02, 0.12]; p < 0.001). With one-year hospitalizations, patients with greater than six encounters experienced an 80% decrease in the likelihood of events (p < 0.001). Additionally, patients who had stable housing showed 61% decreased odds of one-year hospitalization (OR [95% CI]: 0.39 [0.21, 0.71]; p = 0.002). Housing status, the number of encounters, diagnosis, and tobacco risk were significantly associated with office follow-ups. Unhoused patients were 33x more likely to experience an office follow-up visit (p< 0.001). Furthermore, patients who needed a device adjustment or management observed a 4.7x increase in the odds of office follow-up visits. Conversely, patients who were high-risk tobacco users showed lower odds of office follow-up visits compared to low-tobacco-risk users (p = 0.004). 

**Table 2:** Association Between Substance Use (SUD), Selected Demographics, and Clinical Covariates With the Odds of 30-Day, 90-Day, and One-Year Re-Hospitalizations and Office Follow-Up Within a Matched Cohort

Variables	30-Day Hospitalization		90-Day Hospitalization		1-Year Hospitalization		Office Follow-Up	
	OR (95% CI)	p-value	OR (95% CI)	p-value	OR (95% CI)	p-value	OR (95% CI)	p-value
SUD								
No	REF	0.37	REF	0.018	REF	0.5	REF	0.97
Yes	1.32 (0.73, 2.38)		1.44 (0.84, 2.47)		0.86 (0.55, 1.35)		1.01 (0.54, 1.89)	
Unhoused								
No	Not Significant	N/A	Not Significant	N/A	REF	0.002	REF	< 0.001
Yes	Not Significant		Not Significant		0.39 (0.21, 0.71)		33.5 (16.5, 67.9)	
Number of Encounters								
< 2	REF		REF		REF		REF	
2 – 6	0.15 (0.07, 0.34)	< 0.001	0.27 (0.12, 0.58)	0.001	0.56 (0.26, 1.22)	0.14	4.51 (0.49, 41.7)	0.18
7 – 20	0.02 (0.005, 0.06)	< 0.001	0.04 (0.02, 0.12)	< 0.001	0.19 (0.08, 0.43)	< 0.001	8.58 (0.93, 79.1)	0.058
> 20	0.005 (0.001, 0.04)	< 0.001	0.005 (0.006, 0.05)	< 0.001	0.06 (0.02, 0.17)	< 0.001	32.8 (3.48, 308.2)	0.002
Diagnosis								
None	Not Significant	N/A	Not Significant	N/A	Not Significant	N/A	REF	
Any Heart Disease	Not Significant		Not Significant		Not Significant		1.36 (0.67, 2.77)	0.39
Any Device Malfunction	Not Significant		Not Significant		Not Significant		0.50 (0.09, 2.56)	0.41
Device Adjustments/Management	Not Significant		Not Significant		Not Significant		4.79 (1.47, 15.6)	0.009
Infection	Not Significant		Not Significant		Not Significant		N/A	N/A
Tobacco Risk								
Low	Not Significant	N/A	Not Significant	N/A	Not Significant	N/A	REF	
Medium	Not Significant		Not Significant		Not Significant		1.21 (0.36, 4.04)	0.76
High	Not Significant		Not Significant		Not Significant		0.15 (0.04, 0.54)	0.004
Missing	Not Significant		Not Significant		Not Significant		0.25 (0.09, 0.69)	0.008

## Discussion

Substance use is a complex issue influenced by various social determinants of health and co-morbidities, both of which can influence clinical decision-making regarding effective treatment plans for patients reporting active substance use. It has been historically demonstrated that a large portion of patients with heart failure and active substance use leave care against medical advice, which can lead to questions around their ability to follow up appropriately [9]. Therefore, it has been hypothesized that there may be clinical apprehension around CIED implantation in patients with active substance use, influenced by perceived challenges around follow-up adherence and device management, potentially predisposing this population to adverse outcomes [2]. However, data evaluating outcomes among CIED patients with active substance use remains limited. In this study, no significant differences were observed in the rates of rehospitalizations and follow-up adherence between CIED patients with and without current substance use. Similarly, patients with active substance use were not found to be at an increased risk for device infections or malfunctions compared to patients without active substance use.

It is important to note that the retrospective design of this study limits the findings to observational associations and that other factors can contribute to the decision-making process for CIED implantation in patients with active substance use. For example, previous studies have suggested that CIEDs may offer limited benefit in this population. In a retrospective analysis, it was demonstrated that ventricular arrhythmias were uncommon in patients with toxic dilated cardiomyopathy from substance use and that there was no observable mortality benefit to prophylactic ICD implantation in these patients [4]. In addition, it has also been demonstrated that cocaine users receiving ICDs experienced an increase in defibrillation threshold compared to control patients, indicating an increased risk for repeated shocks [2,10,11]. Other modes of treatment, such as pharmacological methods, have also been proposed as ways to treat arrhythmias in patients with active substance use. Beta-adrenergic antagonists, alpha-adrenergic antagonists, and calcium channel blockers have all been used in patients with reported cocaine use; however, treatment remains controversial due to a lack of consistent evidence of patient benefit [2]. Beta-adrenergic antagonists are particularly controversial in cocaine users due to the potential for unopposed alpha-adrenergic activation [2]. Recent studies, however, have demonstrated that this effect is uncommon [2].

Overall, the lack of statistical difference in rehospitalizations, follow-ups, or increased incidence of device-related complications in patients with active substance use seen in this study provides observational insight into the outcomes of CIED recipients in this population and adds to the growing body of literature surrounding this multifaceted clinical challenge.

In addition to conclusions around substance use status and CIEDs, this study also demonstrated trends around ethnicity. In the unmatched analysis, Hispanic/Latino patients were less likely to have rehospitalizations across 30 days, 90 days, and one year compared to non-Hispanic/Latino patients. Historically, the Hispanic/Latino community has been disproportionately affected by cardiovascular disease [12,13]. Evidence from one study demonstrated that Hispanic patients have had higher rates of 30-day rehospitalizations for heart failure compared to White patients, with Hispanic-serving hospitals showing an overall higher rate of rehospitalizations than non-Hispanic-serving hospitals [14]. It has also been historically demonstrated that despite there being an increase in overall cardiology follow-ups between 2010 and 2019, Hispanic/Latino patients experienced worsened disparities in follow-ups, specifically following acute myocardial infarction and heart failure [15]. In this study, the unmatched analysis demonstrated that Hispanic/Latino patients had an increase in office follow-ups compared to non-Hispanic/Latino patients; however, this value did not reach statistical significance (p=0.053). Following the propensity score match, ethnicity was no longer a significant factor in re-hospitalizations or office follow-ups; therefore, potential confounders could have influenced the trends observed in the unmatched analysis.

One of the more notable findings in this study is that individuals experiencing homelessness were 23-fold (unmatched analysis) and 33-fold (matched analysis) more likely to have office follow-ups compared to housed individuals. This finding is significant considering the difficulty of obtaining reliable healthcare in this population [16]. One hypothesis for this observation is that individuals experiencing homelessness are receiving assistance in establishing health resources and transportation for follow-up after hospital discharge through the Valleywise Health partnership with Central Arizona Shelter Services [17]. Further information is required to develop other possibilities for this finding. Additionally, overall hospitalizations amongst unhoused patients are increasing [16], with those experiencing acute myocardial infarction facing higher 30-day readmission rates compared to housed patients [18]. This study demonstrated, in the unmatched analysis, no difference in 30-day, 90-day, or one-year rehospitalizations in unhoused patients compared to housed patients. However, in the matched analysis, patients with stable housing showed 61% decreased odds of one-year hospitalization compared to unhoused patients.

This study is not without its limitations. Due to the retrospective nature of the study, causation cannot be determined. Additionally, some conclusions and interpretations have been based on the analysis of the unmatched data, which can introduce potential confounders. Potential confounders that were not accounted for in this study include duration and type of substance use, severity of comorbidities, socioeconomic status, other social determinants of health, and adherence to medical advice, which could have influenced the observed results. The reliance on ICD-10 codes for diagnostic categorization may introduce misclassification bias. Furthermore, the single hospital setting of this study limits the generalizability of its findings to wider populations.

Future studies involve repeating this analysis within national databases to determine if similar trends exist on a national level.

## Conclusions

This study demonstrated a lack of statistical difference in rehospitalizations and follow-ups between patients with CIEDs with and without current substance use. This study also demonstrated that patients with CIEDs and current substance use were not at an increased risk for device infections or malfunctions compared to patients without current substance use. Further studies are necessary with larger populations to validate these conclusions.
